# Multisite joint pain in older Australian women is associated with poorer psychosocial health and greater medication use

**DOI:** 10.1186/s12998-018-0224-9

**Published:** 2019-02-12

**Authors:** Katie de Luca, Arnold Wong, Andreas Eklund, Matthew Fernandez, Julie Ellen Byles, Lynne Parkinson, Manuela Loureiro Ferreira, Jan Hartvigsen

**Affiliations:** 1 Research Centre for Generational Health and Ageing, University of Newcastle, Callaghan, NSW Australia; 2 Department of Chiropractic, Faculty of Science and Engineering, Macquarie University, Sydney, NSW 2109 Australia; 3 Chiropractic Academy for Research Leadership (CARL), Sydney, Australia; 4 Department of Rehabilitation Sciences, The Hong Kong Polytechnic University, Hong Kong, SAR China; 5 Unit of Intervention and Implementation Research for Worker Health, The Institute of Environmental Medicine, Karolinska Institutet, Stockholm, Sweden; 6 Central Queensland University, LEAP Centre, Gladstone, QLD Australia; 7 Institute for Bone and Joint Research, The Kolling Institute, University of Sydney, Sydney, NSW Australia; 8 Department of Sports Science and Clinical Biomechanics, University of Southern Denmark, Odense, Denmark; 9 Nordic Institute of Chiropractic and Clinical Biomechanics, Odense, Denmark

**Keywords:** Pain, Musculoskeletal pain, Arthralgia, Aging, women’s health, Quality of life, Epidemiology

## Abstract

**Background:**

Musculoskeletal pain frequently occurs in more than one body region, with up to 80% of adults reporting more than one joint pain site in the last 12 months. Older people and females are known to be more susceptible to multiple joint pain sites, however the association of multisite joint pain with physical and psychosocial functions in this population are unknown.

**Methods:**

Cross-sectional data from 579 women were analyzed. Women were asked “Which of your joints have been troublesome on most days of the past month?” Pain qualities were measured using the McGill Pain Questionnaire (Short Form) and PainDETECT, and health was assessed using the SF-36 and sociodemographic variables. Statistical analysis using generalized ordinal logistic regression included comparison of three joint pain groups: no joint pain, 1–4 sites of joint pain and ≥ 5 sites of joint pain.

**Results:**

Two thirds of respondents had multisite pain (>1 site), and one third had ≥5 joint pain sites. Compared to women with fewer joint pain sites, women with >5 joint pain sites (multisite joint pain) had significantly poorer physical and emotional health-related quality of life, more severe pain, a higher probability of neuropathic pain, and a longer duration of pain. More than half of women in the multisite joint pain group were still employed, statistically significantly more than women with no joint pain. In the final model, pain duration, the number of medications, pain intensity (discomforting and distressing) and the physical component of health-related quality of life were significantly associated with increased number of joint pain sites.

**Conclusions:**

Over one-third of older women in our sample had >5 painful joints in the last month. These women demonstrated significantly poorer psychosocial health, and increased medication use, than women with no or fewer sites of joint pain. Many women with multisite joint pain were still in the workforce, even when nearing retirement age. This study has important implications for future research into musculoskeletal pain, particularly in regards to womens health and wellbeing, and for clinical practice where there should be increased awareness of the implications of concurrent, multisite joint pain.

## Background

Musculoskeletal pain is the most common cause of disability globally [1] with increasing disability of low back pain primarily due to population growth and aging [2]. Musculoskeletal pain frequently occurs in more than one body region [3], with up to 80% of adults reporting more than one joint pain site in the last 12 months [4]. People with multisite joint pain (MSJP) are also prone to suboptimal clinical outcomes [5–8], greater healthcare utilization [9], reduced work productivity [10], poorer health status [11] and reduced activities of daily living [12], than those with single site joint pain. Additionally, MSJP is known to be associated with greater physical impairment and psychological distress [13], impaired sleep quality [14] and poor prognosis regardless of treatments [13].

Older people and females are consistently found to be more susceptible to widespread pain [4, 12, 15, 16]. Individuals above the age of 65 years are more likely to present with moderate to severe chronic episodes of spinal pain, and their pain is more likely to be incapacitating when compared to younger adults [17, 18]. Data from the 2011 National Health and Aging Trends Study showed that women aged ≥65 years had a higher prevalence of pain (57.7% vs 46.7%), higher prevalence at each anatomic site and a greater total number of pain sites in comparison to men (i.e., 22.3% of women had ≥4 sites of pain compared to 13.4% of men) [19]. Whilst this study concluded that MSJP significantly compromised measured physical performance and self-reported physical function of older adults [19], the associations of MSJP intensity or duration on physical and psychosocial functions, as well as neuropathic pain in community-dwelling older adults was uncertain.

The aim of this study was to investigate associations between the number of joint pain sites and self-rated health-related quality of life, pain characteristics, and sociodemographic variables in a representative sample of community-dwelling, older Australian women.

## Methods

### Study design and participants

The Australian Longitudinal Study on Women’s Health (ALSWH) is a longitudinal population-based survey that has been studying the health of a national sample of over 40,000 Australian women since 1996 [20, 21]. Women are sampled from four cohorts (new-young, born 1989–95; young, born 1973–1978; mid-age, born 1946–1951; and older, born 1921–1926). ALSWH surveys are sent to each cohort triennially, with surveys staggered for each cohort over 3 years. ALSWH participants are often invited to answer additional sub-study surveys between the major triennial surveys.

From March to November 2012, we collected data from a cross-sectional sub study survey that explored the characteristics of pain in older women [22]. The sub-study survey involved 700 community-dwelling women from the mid-age, born 1946–1951, cohort. The sub study deliberately oversampled women with arthritis: the survey was sent to 350 random women who answered ‘yes’ to “arthritis/rheumatism”, for the question “In the past THREE years, have you been diagnosed or treated for:” in Survey 3 (2001) or Survey 4 (2004) and 350 random women who have never reported any form of arthritis in Surveys 3–6 (2001–2010). Our study conforms to the appropriate reporting guidelines for observational studies (cross-sectional studies) in accordance with the STROBE (STrengthening the Reporting of OBservational studies in Epidemiology) guidelines [23]. This study has been approved by the Human Research Ethics Committee of the University of Newcastle; Approval number: H-2012-0144.

### Primary outcome measures

The primary outcome measures were the location and number of joint pain sites. Women marked on a whole-body homunculus, “Which of your joints have been troublesome (painful, aching, swollen or stiff) on most days of the past month? (Please mark ALL boxes that are applicable to each joint.)”. The total number of joint pain sites (range 0–22) was categorized into three groups: no joint pain (reference group), 1–4 sites of joint pain (some joint pain) and 5–22 sites of joint pain (many joint pain sites). This classification method is consistent with cut-points used in other studies [24, 25].

### Secondary outcomes measures

The secondary outcome measures for analyses were pain characteristics, health-related quality of life scores and sociodemographic and health behaviour variables.

### Pain characteristics

Pain characteristics were measured as self-reported duration of pain (in months), and the present pain intensity (PPI Scale) of the McGill Pain Questionnaire (Short Form) (SF-MPQ) [26]. The SF-MPQ is the most widely used, accepted and comprehensive assessment of the pain experience in older persons [27]. Neuropathic pain is defined as “pain arising as a direct consequence of a lesion or disease affecting the somatosensory system” [28], and in this study the PainDETECT was used as a self-reported screening tool for neuropathic pain. It includes three 11-point numerical rating scales on current pain, as well as strongest and average pain in the last month. Nine items relate to sensory descriptors and the temporal and spatial characteristics of pain. Scores ≤12 indicate that a neuropathic component is unlikely, and scores ≥19 indicate likely neuropathic pain; scores between 13 and 18 reflect a possible/ambiguous neuropathic pain component [29]. In previous research, a modified painDETECT score cut-point of ≤12 was used in patients with knee osteoarthritis [30] to reflect the mixed neuropathic/nociceptive pain mechanism in arthritis [31, 32]. As such, participants’ responses in this study were dichotomised into the presence or absence of neuropathic-like pain based on the screening cut-off value of 12.

### Health-related quality of life

Health-related quality of life was assessed using the Medical Outcomes Study: 36 Item Short Form Survey (SF-36); a well-validated health profile that has been used in a broad variety of patient populations for its brevity, ability to discriminate among disease states, and acceptability to patients [33]. The SF-36 assesses eight different domains of health and responses from the 36 individual items that can be aggregated into a physical component summary (PCS) and mental component summary (MCS) score relative to population norms [34]. The scales are scored on a range of 0–100 with norms at 50 and a higher score representing better physical function, better health, and better physical and mental health.

### Sociodemographic and health behaviour variables

The following sociodemographic and health behaviour characteristics were evaluated using linked data from Survey 6 (2010) of the mid-age, born 1946–1951, cohort, area of residence (‘urban’ or ‘rural’ according to the Rural Remote and Metropolitan Areas classification system) [35]; marital status (married/de facto or separated/divorced/widowed/single); smoking status (‘never/ex-smoker’ or ‘current smoker’) [36]; alcohol status (‘non/rare-drinker’ ‘low risk/high risk’) [37] and participation in the labour force (employed or not employed). Linked health data included Body Mass Index (BMI) (aggregated into three categories: ‘underweight/normal’, ‘overweight’ and ‘obese’) [38] and self-reported number of prescribed medications. As all respondents were women between a specific age range, gender and age were not included as confounders.

### Statistical analysis

The participants’ characteristics were compared among the three groups defined by the number of pain joint pain sites, using chi-squared tests for categorical variables, or one-way ANOVA for continuous variables. Univariate ordinal logistic regression was used to assess the association between joint pain sites (comparing no joint pain to the combination of some and many and comparing the combination of no joint pain and some to many; where the proportional odds assumption is satisfied, and the coefficient is the same between levels) and pain, health, and sociodemographic variables. The conservative level *p* < 0.25 was chosen as the screening criterion for variable inclusion into the multivariable ordinal logistic regression model. For multivariate analyses, the assumption of proportional odds was not satisfied for standard ordinal logistic regression. Therefore, generalized ordinal logistic regression, which relaxes the proportional odds assumption and allows the effects of the explanatory variables to vary with the point at which the categories of the dependent variable are dichotomized, was used to describe associations. Positive coefficients mean that higher values on the covariates make higher values on the dependent variable (i.e. number of pain sites) more likely. A parsimonious model was obtained using a stepwise backward elimination approach. The final reduced model provided coefficients for the change in odds of being in a higher joint pain category for each variable and adjusted for other predictors in the model. Statistical significance was set at *p* < 0.05, with the corresponding odds ratios (OR) and 95% confidence intervals (CI’s) reported. All analyses were conducted using statistical program STATA 12.0 (StataCorp LP, College Station, TX, USA).

## Results

**Fig. 1:**
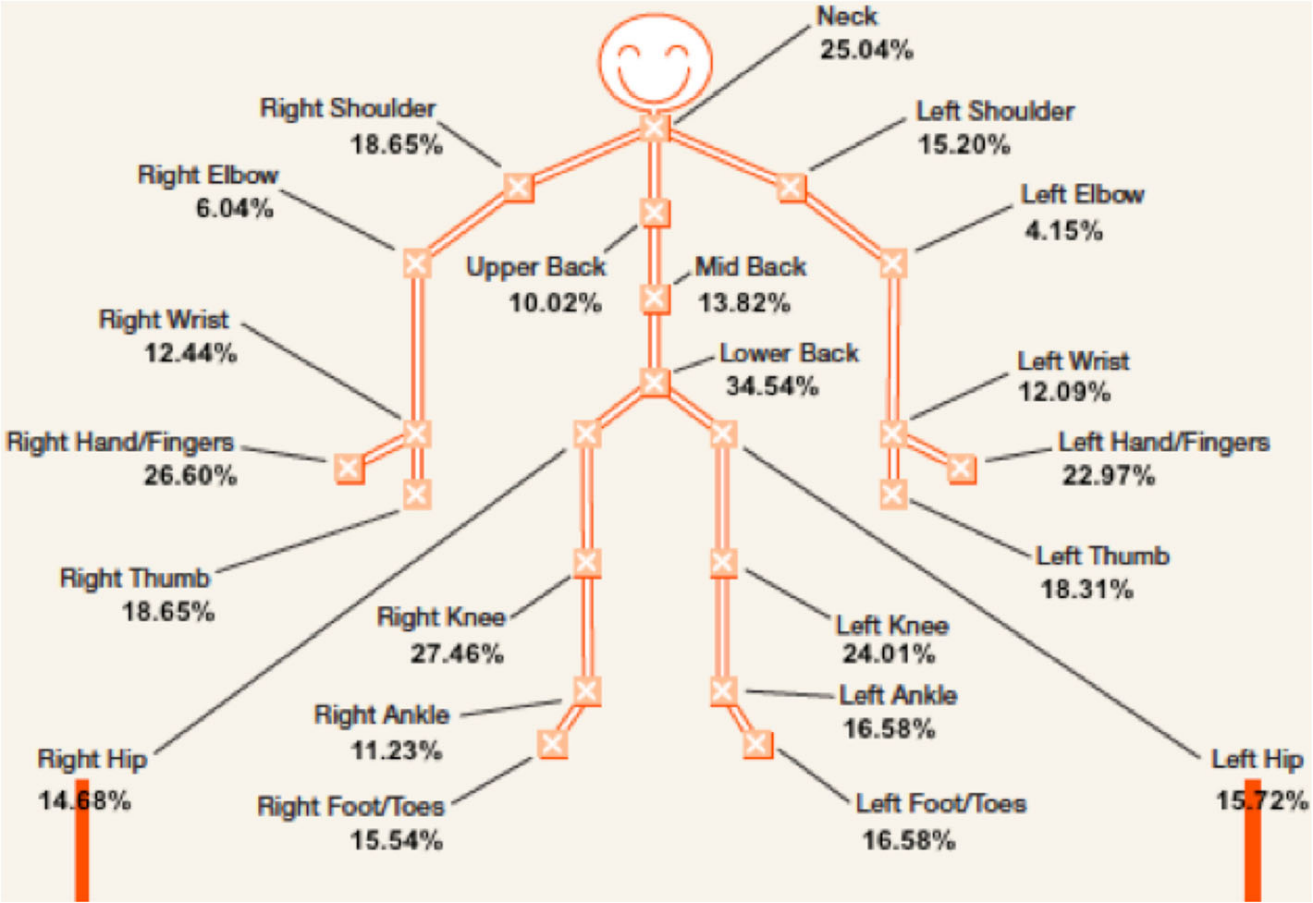
The location and prevalence of 22 joint pain sites, painful during the last month, self-reported by older Australia women and recorded on a whole-body homunculus

**Fig. 2:**
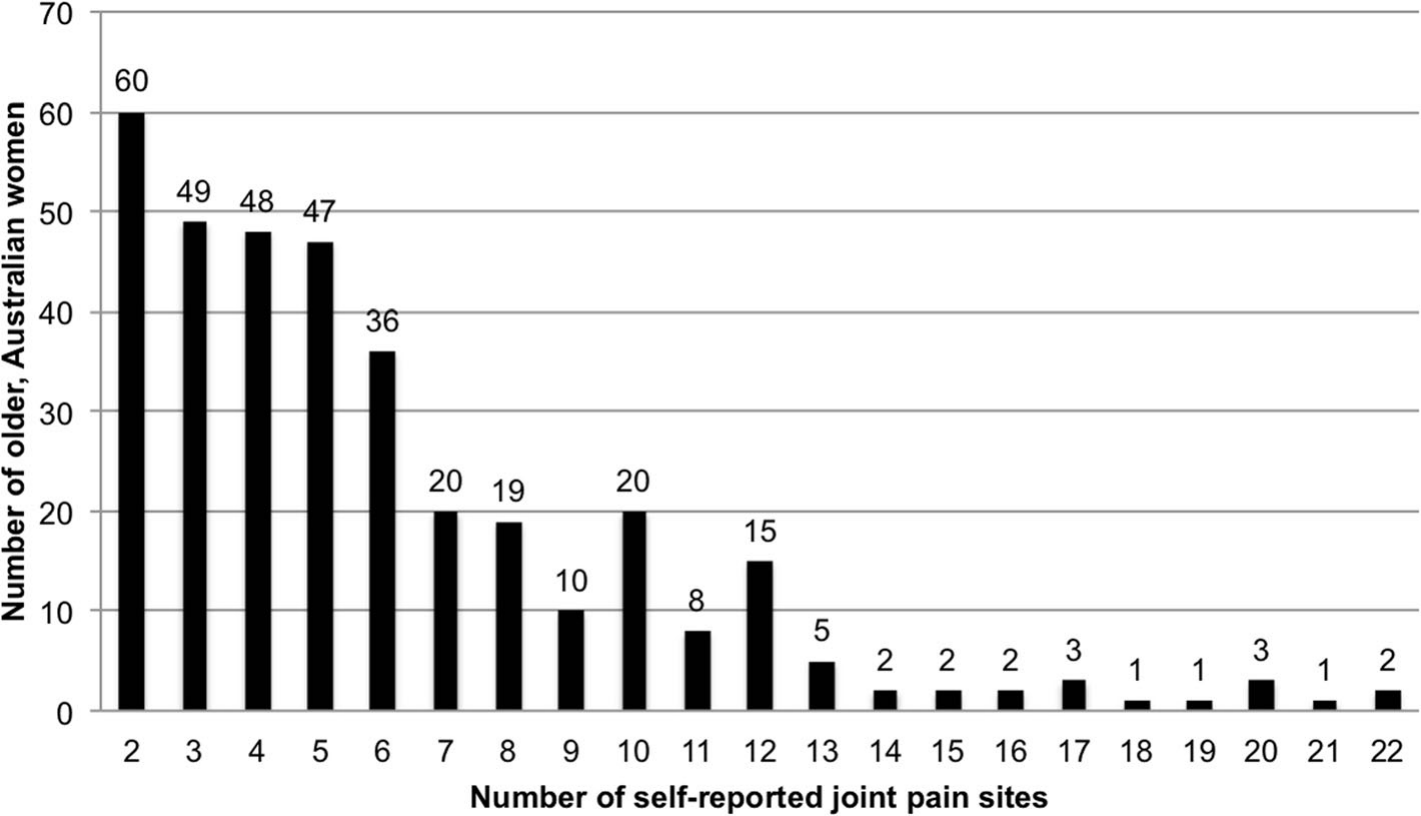
The frequency of the number of self-reported multiple joint pain sites (> 1) during the last month, by 354 older, community dwelling Australian women

Of the 700 women invited to participate in the sub-study survey, 579 women (mean age 64.6 ± 1.5 years) consented to participate and returned surveys (82.7% response rate). Of these, 177 women (30.6%) reported that they had never had troublesome joints/no joint pain in the last month, 48 women (8.3%) reported having one location of joint pain, and 354 women (61.1%) had pain affecting more than one joint (mean 3.8 ± 4.2 joint pain sites). Classifying women into groups, 205 women (35.4%) had “some joint pain” (1–4 sites) and 198 women (34.0%) had “many joint pains” (5–22 sites). The location and prevalence of each self-reported joint pain site is shown in Fig. 1. The low back (34.5%), right knee (27.5%), right hand (26.6%) and the neck (25.0%) were the most common sites of joint pain. Figure 2 displays the frequency of the number of self-reported multiple joint pain sites (> 1) during the last month.

Table 1 shows the differences in pain characteristics, health-related quality of life scores and sociodemographic and health behaviour variables between the three groups. The proportion of women employed was the highest in the group with MSJP, with 51.0% of these women employed compared to 39.4% in the no joint pain group. Women in the MSJP group also had the highest proportion of obesity (34.5%), compared to some joint pain (28.8%) and no joint pain (17.5%). The proportion of women screened as having possible neuropathic pain was the highest in the MSJP group (31.3% vs 15.6% (some joint pain); and 31.3% vs 16.7% (no joint pain), *p* = 0.02). Similarly, the proportion of women with distressing/horrible/excruciating pain was the highest in the MSJP group (19.5% vs 3.5% (some joint pain) and 19.5% vs 5.9% (no joint pain), *p* < 0.0001). Women with some joint pain and many joint pain sites had a significantly lower mean physical and mental quality of life scores than women with no joint pain (SF-36 PCS 50.0 ± 10.0 vs. 47.6 ± 7.9 vs 38.0 ± 11.0, *p* < 0.0001; and SF-36 MCS 54.2 ± 7.2 vs. 52.4 ± 9.1 vs 50.0 ± 11.1, *p* < 0.0001).

**Table 1:** Sample characteristics of 579 community-dwelling older women, and differences in pain, health and sociodemographic characteristics between women with none, some joint pain and many joint pain sites

	Total (*n* = 579)	No joint pain (0 sites) (*n* = 177)	Some joint pain (1–4 sites) (*n* = 205)	Multisite joint pain (5–22 sites) (*n* = 197)	*p*
Mean age (mean; ±SD)	64.6 (1.5)	64.5 (1.5)	64.6 (1.4)	64.6 (1.5)	0.46
Residence, no. live rural (%)	359 (62.0)	100 (65.5)	126 (61.5)	133 (67.5)	0.89
Marital, no. married/defacto (%)	467 (80.6)	147 (83.1)	168 (82.0)	152 (77.2)	2.99
Employed (%)	249 (43.3)	69 (39.4)	80 (39.2)	100 (51.0)	0.03
Smoking status (never/ex-smoker) (%)	535 (92.4)	161 (91.0)	195 (95.1)	179 (91.0)	0.12
Alcohol status (non/rare-drinker) (%)	208 (38.0)	81 (47.4)	55 (28.5)	72 (39.3)	< 0.001
BMI					
Healthy / underweight (%)	211 (36.4)	85 (48.0)	75 (36.6)	51 (25.9)	< 0.001
Overweight (%)	210 (36.3)	61 (34.5)	71 (34.6)	78 (39.6)	
Obese (%)	158 (27.3)	31 (17.5)	59 (28.8)	68 (34.5)	
SF-36 physical component scale (mean; ±SD)	45.0 (10.9)	50 (10.0)	47.6 (7.9)	38 (11.0)	< 0.001
SF-36 mental component scale (mean; ±SD)	52.1 (9.5)	54.2 (7.2)	52.4 (9.1)	50.0 (11.1)	< 0.001
Duration of joint pain (months) (mean; ±SD)	118.8 (121.2)	90.9 (118.6)	85.5 (99.0)	154.4 (130.9)	< 0.01
painDETECT (neuropathic-like pain) (%)	63 (24.4)	4 (16.7)	14 (15.6)	45 (31.3)	0.02
Present Pain Intensity (mean; ±SD)					
Mild/Moderate	185 (43.4)	17 (50.0)	125 (61.9)	43 (22.6)	< 0.001
Discomforting	195 (45.8)	15 (44.1)	70 (34.7)	110 (57.9)	
Distressing/Horrible/Excruciating	46 (10.8)	2 (5.9)	7 (3.5)	37 (19.5)	
Number of prescribed medications (mean; ±SD)	4.3 (3.3)	3.4 (3.1)	3.9 (2.8)	5.6 (3.7)	< 0.001

*Abbreviations*: *BMI* body mass index, *SD* standard deviation, *SF-36 PCS* Medical Outcomes Study 36 Item Short Form Survey Physical component scale, *SF3–6 MCS* Medical Outcomes Study 36 Item Short Form Survey Mental health component scale

Older women with MSJP were more likely to live rurally, be overweight or obese, have more severe pain, have poorer physical and emotional health-related quality of life, have possible neuropathic pain and use more medications (Table 2). In the final model (Table 3), the proportional odds assumption was maintained for the number of medications and duration of joint pain, whilst the proportional odds assumption was relaxed for present pain intensity and SF-36 physical component scale. In this final model, pain duration (95% CI 1.00, 1.00; *p* < 0.01), the number of medications (95% CI 1.00, 1.16, 9.18; *p* < 0.05), pain intensity (discomforting 95% CI 1.41, 3.93; *p* < 0.01; distressing (95% CI 1.74, 14.78; *p* < 0.01) and the physical component of health-related quality of life (95% CI 0.92, 0.98; *p* < 0.001) were significantly associated with a higher number of joint pain sites (Table 3).

**Table 2:** Univariate ordinal logistic regression analysis of the variables associated with no joint pain, some and many joint pain sites across pain, health, and sociodemographic variables

Variable	OR (95% CI)	*P-*value
Area of residence		
Urban	1	
Rural	1.4 (1.0–1.9)	0.03
Smoking		
Never smoked/ex smoker	1	
Current smoker	1.0 (0.6–1.9)	0.90
Marital status		
Married/de facto	1	
Separated/divorced/widowed/single	1.3 (0.9–2.0)	0.14
Employment		
Employed	1	
Not employed	0.7 (0.5–0.9)	0.02
BMI		
Normal / Underweight	1	
Overweight	1.9 (1.3–2.7)	< 0.01
Obese	2.6 (1.8–3.9)	< 0.001
Duration of joint pain	1.0 (1.0–1.0)	< 0.001
Present Pain Intensity		
Mild/Moderate	1	
Discomforting	3.4 (2.2–5.1)	< 0.001
Distressing/Horrible/Excruciating	10.9 (5.0–24.1)	< 0.001
SF-36 PCS	0.9 (0.9–0.9)	< 0.001
SF-36 MCS	1.0 (0.9–1.0)	< 0.001
Neuropathic pain		
No	1	
Yes	2.3 (1.3–4.3)	0.01
Number of medications	1.2 (1.1–1.2)	< 0.001

**Table 3:** Generalised ordinal logistic regression analysis of the variables associated with none, some and many joint pain sites across pain, health, and sociodemographic variables

	No joint pain *compared to* multisite joint pain (some and many joint pain sites)			No joint pain and some joint pain *compared to* many joint pain sites		
	Odds Ratio	95% CI	*p* value	Odds Ratio	95% CI	*p* value
Number of prescribed medications	1.08	1.00, 1.16	0.05	1.08	1.00, 1.16	0.05
Duration of joint pain	1.00	1.00, 1.00	< 0.01	1.00	1.00, 1.00	< 0.01
Present Pain Intensity						
Discomfort	0.74	0.3, 1.84	0.52	2.35	1.41, 3.93	< 0.001
Distressing/Horrible/Excruciating	5.09	1.75, 14.78	< 0.01	5.09	1.75, 14.78	< 0.01
SF-36 physical component scale	1.97	0.98, 1.01	0.15	0.95	0.92, 0.98	< 0.001

*Abbreviations*: *CI* confidence interval, *SF-36 PCS* Medical Outcomes Study 36 Item Short Form Survey Physical component scale

In the final reduced model of generalised ordinal logistic regression, coefficients for the change in odds of having multisite joint pain (comparing no joint pain to the combination of some and many joint pain sites (column two); and being in a higher joint pain category (comparing the combination of no joint pain and some joint pain sites to many joint pain sites (column three)) for each predictor and adjusted for other predictors in the model are shown

## Discussion

This study shows that in a sample of community-dwelling, older Australian women, MSJP is common, with more than two thirds of women reporting more than one site of joint pain. The high percentage of women with MSJP is consistent with previous population-based studies [12, 24, 39]. A longer duration and higher intensity of joint pain, as well as poor physical quality of life and more medications are associated with a higher number of joint pain sites. From our study, it is noteworthy that the odds of having “Distressing/Horrible/Excruciating” pain are almost 11 times higher for older women with MSJP, highlighting the experience of severe joint pain in this population. Older women with MSJP demonstrated poorer physical and mental quality of life as compared to those without joint pain. It is well established that psychological factors are implicated in chronic pain with depression, anxiety, fear and poor sleep associated with low back pain [40], neck pain [41], widespread pain (fibromyalgia) [42] and osteoarthritis [43]. Although mental quality of life was not associated with a higher number of joint pain sites in the final generalised ordinal logistic regression models, univariate analysis shows that further research is warranted; specifically, in exploring the relationship between depression, anxiety, mood and fear in people with MSJP.

In this study, low back pain had the highest prevalence of joint pain in older women (35%). This is less than the reported low back pain prevalence in older people with chronic MSJP (62%) [44], probably reflecting the difference between the community-dwelling and primary care referral samples. Recent research highlights the inappropriate use of imaging, opioids, spinal injections and surgery - all are potentially harmful and ineffective in reducing low back pain related disability [45]. Our study has shown that a higher level of prescription medication usage was associated with MSJP. Prescribed medications in our sample may not be related only to painful joints, however painful joints [46, 47] are associated with a greater prevalence of psychological comorbidities (e.g. depression or anxiety) and sleep disturbances [48, 49] that do often require medication prescription. Additionally, a higher number of comorbidities (diabetes, pulmonary disease, cardiovascular disease and obesity) is associated with a higher risk of spinal pain [50], a relationship, which may also explain the higher use of prescribed medications. Opioids are frequently prescribed to older people with MSJP [44], with older people 70% more likely to receive a prescription for pain-killers. Unfortunately, older people are 50% less likely to be advised about manual therapy and exercise, compared to younger patients [51]. Older people with joint pain are also at risk of polypharmacy and the use of non-pharmacological interventions are disparate [52], suggesting the management of joint pain is sub optimal [53]. Previous research has shown low back pain is associated with patterns of pain that include both very high and high probabilities of pain across the entire body [3], so possibly, the number of joint pain sites may be a better measure for musculoskeletal health than complaints at any single site. Future research should look to implement best practice in the redesign of clinical pathways to minimize the burden of concurrent, multiple sites of joint pain.

As older women with MSJP had twice the risk of having neuropathic-like pain than older women without joint pain, neurological wind up may explain their heightened pain perception to innocuous stimuli [54, 55]. For women in our study who had MSJP, pain intensity increased in a dose response like relationship with an increasing number of joint pain sites. This reveals that mechanisms within MSJP may include the manifestation of abnormal sensory processing within the central nervous system due to dysregulation of the nociceptive pain pathway [54].

More than half of the women (aged 61–66 years) with MSJP were still in the workforce, which was significantly higher than women with some or no joint pain. Women in the MSJP group also had higher pain intensity, which has been shown to be associated with the onset of work productivity loss [56]. We do not know the level of workforce participation (part-time or full-time for example), but this study highlights that older women at work have more joint pain, and this has implications for the management of workers who suffer from joint pain. With the onset of work restriction in employed adults with lower limb joint pain, involuntary exclusion from the labour force is significant associated with being older and being more depressed [57]. Management strategies to improve function may have an indirect effect by decreasing the impact of pain on work productivity, which is especially important, as significant pain reduction is often difficult to achieve. Furthermore, there is a need to investigate workplace exposures and modifications for people at work, and in pain, in order to promote healthy workplaces and support people to work later into life. Future qualitative studies should explore the employment experience of women with MSJP, particularly with regards to workforce participation, presenteeism and pain management interventions.

The current study has several limitations. A consideration for the interpretation of this work is the sampling of older women with arthritis from ALSWH. For the purpose of this study, 350 women who had reported arthritis/rheumatism in 2001 or 2004 were sampled, and therefore the prevalence may have been higher compared to a cross-sectional study from the general population. Previous research has shown the contemporaneous severity of symptoms in this population of Australian women; from 46% of women who reported arthritis on at least one survey, half later reported not having arthritis [58]. Variations in the reporting of arthritis in ALWSH surveys prior to 2012, led to the methodological considerations of oversampling in this cross-sectional survey. The cross-sectional study design also prevents the identification of causal relationships. Future longitudinal research is therefore warranted to identify predictors for MSJP. The arbitrary cut-points of joint pain sites may be seen as a limitation of the study, however a thorough search of the literature did not reveal standardized definition of MSJP, and the two cut-points chosen were consistent with recent MSJP research [24, 25].

Our study is strengthened by sampling from a large, nationally representative population-based cohort of Australia women (ALSWH). The recall period of joint pain was within the last month, which minimized self-report recall bias regarding pain and function. This study also utilized comprehensive measures of pain suitable for cross-sectional surveys [59], including the SF-36, McGill Pain Questionnaire and painDETECT.

## Conclusion

Over one-third of older women in our sample had > 5 painful joints in the last month. These women demonstrated significantly poorer psychosocial health, and increased medication use, than women with no or fewer sites of joint pain. Many women with multisite joint pain were still in the workforce, even when nearing retirement age. This reveals a need for an exploration of the factors keeping women at work, whilst in pain, and providing appropriate models of care to minimize work-related disability. This study has important implications for future research into musculoskeletal pain, particularly in regards to womens health and wellbeing, and for clinical practice where there should be increased awareness of the implications of concurrent, multisite joint pain.

## Abbreviations

ALSWH: Australian Longitudinal Study on Women’s Health
BMI: Body mass index
MCS: Mental component summary of the Medical Outcomes Study: 36 Item Short Form Survey
MSJP: Multisite joint pain
PCS: Physical component summary of the Medical Outcomes Study: 36 Item Short Form Survey
PPI: Present pain intensity scale of the McGill Pain Questionnaire (Short Form)
SF-36: Medical Outcomes Study: 36 Item Short Form Survey
SF-MPQ: McGill Pain Questionnaire (Short Form)
